# Body Image and Psychopathology in Men Over 65: Is It Time to Revise BMI Cutoffs?

**DOI:** 10.1002/brb3.71091

**Published:** 2025-11-19

**Authors:** Alfonso Martone, Silvia Tempia Valenta, Federica Marcolini, Diana De Ronchi, Emilia Manzato, Anna Rita Atti

**Affiliations:** ^1^ Department of Biomedical and Neuromotor Sciences University of Bologna Bologna Italy; ^2^ Doctoral Program of Global Health, Humanitarian Aid and Disaster Medicine Vrije Universiteit Brussel Brussels Belgium; ^3^ Eating Disorders Unit Former University Hospital “St. Anna” Ferrara Italy

**Keywords:** body perception, body satisfaction, body acceptance, body appreciation, geriatric men, older men

## Abstract

**Introduction:**

Body dissatisfaction (BD) and body appreciation (BA) are relevant psychological concerns that affect individuals across various age groups. However, limited research has focused on these issues among older men. The aim of this study is to investigate the prevalence of BD and BA and their relationships with body mass index (BMI) and mental health symptoms in a sample of community‐dwelling Italian men aged 65 and above.

**Methods:**

Participants in this cross‐sectional study (N = 122) completed self‐report measures including the figure rating scale (FRS), the body appreciation scale (BAS‐2), the Geriatric anxiety scale‐short form (GAS‐10), and the Geriatric depression scale‐short form (GDS‐SF). The Kruskal‐Wallis test was used to examine differences between groups, and the Mann‐Whitney U test was applied for pairwise comparisons.

**Results:**

45.1% of participants experienced some level of BD, with 13.9% demonstrating severe BD, while only 39.3% reported BA. BD was significantly higher in individuals with overweight or obesity. Anxiety and depression symptoms were significantly more common among participants with BD compared to those without. A significant association was also observed between lower BA and increased anxiety, whereas BMI cutoffs did not show significant correlations with BA.

**Conclusion:**

This study highlights the importance of considering BD in older adults. These findings indicate differences in BD across BMI categories, suggesting potential benefits of using age‐specific BMI thresholds (27.27 kg/m^2^ or 28 kg/m^2^) when assessing body image in aging populations. Further research should explore age‐specific differences in body image and their implications for mental health in older adults.

## Graphical abstract

Relationships among body image attitudes, BMI, and psychological symptoms in a sample of older Italian men.

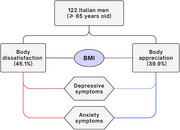

## 1 Introduction

Pierre Bonnier (1861–1918) first introduced the definition of *body schema* to elucidate the human capacity for accurately locating perceived stimuli (Bonnier 2009). In 1935 Paul Ferdinand Schilder, a neuropathologist and psychoanalyst, developed the concept of “body image” as “the picture of our body which we form in our mind,” incorporating various mental aspects such as perceptions, emotions, feelings, and thoughts (Schilder 2013). Over the years, the understanding of body image evolved, and the research distinguished perceptual, affective, cognitive, and behavioral components, influenced by the historical, biological, cultural, and individual factors (Slade 1994; Linden 2004; Grogan 2010). Ledoux and colleagues expanded the definition, describing body image as a multidimensional construct that involves systematic, cognitive, affective, conscious, and unconscious representations (Ledoux et al. 2010; Farias et al. 2018).

Body dissatisfaction (BD) and body appreciation (BA) represent two dimensions of body perception and self‐evaluation. BD refers to a persistent negative evaluation of one's body, characterized by dissatisfaction with body shape or parts, often driven by a desire to align physical appearance with personal or societal standards (Thompson et al. 1999; Halliwell and Dittmar 2006; Grogan 2010; Tiggemann and McCourt 2013). BD can lead to critical self‐scrutiny, lower self‐esteem, and, in severe cases, contribute to mental health issues such as eating disorders (EDs), depression, and anxiety (Stice and Shaw 2002; Grogan 2010). On the other hand, BA encompasses a positive and accepting attitude toward one's body, including respect for its functions and a rejection of unrealistic appearance pressures (Tylka 2019). People with high BA are more likely to feel grateful for their bodies’ abilities, experience higher self‐worth, and demonstrate resilience to societal pressures about appearance (Tylka 2019; Linardon et al. 2022). Together, BD and BA capture the spectrum of body perception, impacting individuals’ mental health and well‐being in distinct ways.

Different studies have confirmed the presence of BD in men across adulthood (Paxton and Phythian 1999; Peat et al. 2008; Campbell and Hausenblas 2009; Roy and Payette 2012; Farias et al. 2018). While weight loss and fear of weight gain are more likely in young women, concerns about body shape and muscle growth are prevalent among young men (Gorrell and Murray 2019). The evolving ideals of the male body, particularly the emphasis on a muscular and sculpted physique in media, contribute to increased levels of body uneasiness (Manzato and Gravina 2018; Frederick et al. 2020). Studies on older men indicate variations in body image concerns, including less desire for muscularity and greater fear of weight gain compared to younger counterparts (Reddy 2013; Song and Oakley 2020; Hooper et al. 2023).

At the same time, the prevalence of obesity in the elderly population is on the rise, intensifying experiences of BD as media‐driven body ideals persist across age groups (Flegal et al. 2012; Jung et al. 2017). Projections suggest that body‐related uneasiness will continue to increase among adults, particularly those with obesity (Vincent and Velkoff 2010). This “normative discontent,” a widespread dissatisfaction with body weight and shape, is increasingly reported even among the elderly (Cuzzolaro 2018). In effect, as individuals age and experience weight gain, the gap between their actual and ideal body image often increase (Tiggemann and McCourt 2013). Older men also face changes such as sarcopenia, a common age‐related decline in muscle mass and function, which affects approximately 10‐16% of the community‐dwelling elderly population and is associated with both physical limitations and increased depression risk. Sarcopenia potentially exacerbates BD as age‐related muscle loss contrasts sharply with the muscular body ideals often promoted by the media (Lv et al. 2024). In this context, body appearance and health issues, such as mobility or independence, can become primary motivators for weight loss in older adults (Jackson et al. 2019).

Di Renzo et al. (2022) questioned the validity of the WHO's body mass index (BMI) ≥ 30 kg/m^2^ threshold for defining obesity, as changes in body composition in older adults, including increased body fat and reduced lean mass, suggest that a BMI of 27.27 kg/m^2^ may be a more accurate predictor of adiposity (Di Renzo et al. 2022). Indeed, in their study, the revised cutoff achieved better sensitivity and specificity for obesity in older adults, suggesting the importance of tailoring BMI thresholds to specific populations (Di Renzo et al. 2022). Similarly, Singh (2024) highlighted that current WHO BMI cutoffs may not accurately predict cardio‐metabolic risks for older adults, recommending new thresholds for underweight, overweight, and obesity leading to an improvement of predictive accuracy for cardio‐metabolic risks, especially in the oldest cohort (Singh and Chattopadhyay 2024). Together, these studies underscore that rigid and universal BMI cutoffs may inadequately address the health needs of aging populations across different countries.

Although societal awareness of body image is increasing, research on body perception in older males remains limited, possibly influenced by stereotypical images of dissatisfaction and age‐related prejudices (Tantleff‐Dunn et al. 2011). In light of this gap, our research aims to (1) estimate the prevalence of BD and BA in a non‐clinical sample of Italian men aged over 65; and (2) explore their relationships with body mass index (BMI), anxiety, and depression symptoms. We hypothesize that (1) older men will show high levels of BD and low levels of BA; and (2) high BD and low BA will be associated with higher BMI, as well as increased symptoms of anxiety and depression symptoms.

## 2 Material and Methods

### 2.1 Study Characteristics

Questionnaires and tests were administered within recreational centers for elders and in residential care facilities, using both paper‐based and digital formats. Prior to the commencement of testing, participants were assured about privacy and data protection, and were required to sign an informed consent form.

Inclusion criteria encompassed male individuals, aged 65 years or older, with adequate proficiency in Italian, and demonstrating the capacity to provide informed consent. Exclusion criteria were poor understanding of the Italian language, ongoing psychotic disorders, or any form of cognitive impairment.

### 2.2 Participants Assessment

An ad hoc structured questionnaire was created to collect participants’ personal histories, including socio‐demographic information such as age, level of educational attainment (ranging from elementary to bachelor's degree), family status (whether living alone, with family members, or with a caregiver), and details regarding physical activity (including type and duration).

Participants reported their current body weight and height, which were used to calculate BMI. BMI categories were initially defined using World Health Organization (WHO) thresholds for underweight, normal weight, overweight, and obesity (World Health Organization (WHO) n.d.). Given critiques regarding the applicability of these thresholds to older adults, since aging is typically associated with increased intra‐abdominal fat and reduced lean body mass (Di Renzo et al. 2022), we also applied alternative, age‐adjusted BMI cutoffs as recommended in the literature (Di Renzo et al. 2022; Singh and Chattopadhyay 2024).

### 2.3 Mental Health Symptoms Assessment

Two self‐report questionnaires were administered to evaluate mental health symptoms: the Geriatric anxiety scale—short form (GAS‐10) (Mueller et al. 2015), and the Geriatric depression scale—short form (GDS‐SF) (Yesavage and Sheikh 1986; Rinaldi et al. 2003).

#### 2.3.1 Geriatric Anxiety Scale‐Short Form (GAS‐10)

The GAS‐10 is a self‐report instrument comprising ten items designed to evaluate anxiety‐related symptoms (Mueller et al. 2015). Respondents rate each item on a 4‐point Likert scale ranging from 0 (never) to 3 (always). The total score is calculated by summing up individual item scores. Participants are categorized based on their total score into those experiencing light (7‐9 points), moderate (10 points), or severe (11 or more) levels of anxiety symptoms. Widely utilized in the geriatric domain, including with neurodegenerative patients, our study employed the version adapted by Ferrari et al. (2017), which is based on the 2015 adaptation derived from the original 30‐item GAS‐10 developed by Mueller et al. (Mueller et al. 2015; Ferrari et al. 2017).

#### 2.3.2 Geriatric Depression Scale‐Short Form (GDS‐SF)

The GDS‐SF is a self‐report assessment tool designed to evaluate depression‐related features through five binary items (yes/no) (Yesavage and Sheikh 1986; Rinaldi et al. 2003). Each affirmative response scores 1 point, while negative responses score 0 points. A total score of 2 or more indicates the presence of depressive traits and suggests a risk for depression diagnosis. The version utilized in our study was the 5‐item adaptation by Rinaldi et al. (Rinaldi et al. 2003), derived from the original 30‐item GDS developed by Yesavage and Sheikh (Yesavage and Sheikh 1986).

### 2.4 Body Perception Assessment

Two self‐report questionnaires were administered to evaluate body perception: the figure rating scale (FRS) (Stunkard et al. 1983) and the body appreciation scale–2 (BAS‐2) (Tylka and Wood‐Barcalow 2015; Casale et al. 2021).

#### 2.4.1 Figure Rating Scale (FRS)

The FRS, colloquially known as “Stunkard Figures” (Stunkard et al. 1983), is a self‐assessment tool for evaluating perceptions of body image dimensions. Participants select from nine male silhouettes with varying body shapes the one they believe most closely resembles their own body and the one representing their ideal body shape. This allows for the assessment of the discrepancy between perceived and desired body shapes (the desired‐perceived gap, DPG), serving as a direct measure of BD. This task enables the assessment of both the desired and perceived body shapes, revealing the disparity between them, known as the DPG. The DPG serves as a direct indicator of BD. Calculating the DPG involves subtracting the number corresponding to the perceived body shape from that representing the ideal body shape. Values exceeding |1| signify a certain degree of BD, indicative of either perceived weight excess or deficiency, further divided into moderate (DPG = |2|) or severe BD (DPG > |2|).

#### 2.4.2 Body Appreciation Scale–2 (BAS‐2)

The BAS‐2 is a 10‐item self‐report scale aimed at assessing BA, with each item rated on a 5‐point Likert scale. Higher scores indicate greater BA (i.e. a positive body image). Cutoff thresholds were established based on quartiles of the total score distribution (Tylka and Wood‐Barcalow 2015; Casale et al. 2021). In the absence of established thresholds for interpreting the BAS‐2, we conducted a normal distribution analysis and established cutoffs as follows: individuals satisfied with their appearance were identified within the last quartile (scoring ≥ 45 points), while unsatisfied participants were classified within the first quartile (total score < 31); in between (total score 31–44), fall those participants with average BA. The scale evidenced construct validity, internal consistency, and temporal stability in older adults (Meneses et al. 2019). In our study, we utilized the Italian adapted version (Casale et al. 2021), derived from the original English version (Tylka and Wood‐Barcalow 2015).

### 2.5 Statistical Analyses

Statistical analyses were performed using IBM SPSS Windows Version 25 and JASP 0.16.4.0. We assessed the normality of variable distributions using the Shapiro‐Wilk test. Descriptive statistics were generated for socio‐demographic factors, including age, educational attainment, and family status, as well as measures of body perception and psychopathology. We then examined differences in BD and BA across three different BMI stratifications utilizing both traditional and updated BMI cutoffs proposed in the literature. For this purpose, the Kruskal‐Wallis test and the Mann‐Whitney U test were applied for standard and alternative BMI stratifications, respectively (since the new proposed cutoffs only identify two categories: body weight within the typical range and overweight/obesity). Finally, we investigated the association between BA and BD and the levels of anxiety and depressive symptoms by using the Kruskal‐Wallis test and the Mann‐Whitney U test, respectively.

## 3 Results

### 3.1 Sample Socio‐Demographic and Clinical Features

Table 1 outlines the characteristics of the sample. Initially, 130 subjects were invited to participate, with three subsequently excluded due to not meeting inclusion criteria and an additional five failing to complete the questionnaire. Consequently, the final sample comprised 122 male subjects, with a median age of 83 years, predominantly from Northern Italy. The median BMI for the sample was 26 kg/m^2^, with 36.1% falling within the typical weight range, 47.5% indicating overweight, 15.6% meeting the criteria for obesity class I, and 0.8% classified as underweight. When the BMI threshold was set at 27.27 kg/m^2^, 43 were individuals with overweight/obesity (35.2%). This percentage decreased to 31.1% (38 participants) when the threshold was adjusted to 28 kg/m^2^. Approximately 4.9% reported a history of anxiety and 2.4% a history of depression.

**TABLE 1:** Sample (N = 122) characterization for socio‐demographic and clinical features.

	Answer	N (%)
Age groups	65–74	82 (67.2)
	75–84	25 (20.5)
	85–94	11 (9)
	Median (Q1–Q3)	83 (79–88)
Educational attainment	None	1 (0.8)
	Primary school	18 (14.8)
	Secondary school	18 (14.8)
	High school	40 (32.8)
	Bachelor's degree	11 (9.0)
	Master/PhD	34 (27.9)
Family status	With family	109 (89.3)
	Alone	12 (9.8)
	With a caregiver	1 (0.8)
BMI	Underweight	1 (0.8)
	Normal weight	44 (36.1)
	Overweight	58 (47.5)
	Obesity first degree	19 (15.6)
	Median (Q1–Q3)	26.0 (24.1–28.4)
BMI (27.27 kg/m^2^ cutoff)	Non‐obesity	79 (64.8)
	Obesity	38 (35.2)
BMI (28 kg/m^2^ cutoff)	Non‐obesity	84 (68.9)
	Obesity	38 (31.1)
Anxiety (GAS‐10)	No anxiety (0–6)	85 (69.7)
	Light anxious symptoms (7–8)	14 (11.5)
	Moderate anxious symptoms (9–10)	7 (5.7)
	Severe anxiety symptoms (> 11)	16 (13.1)
	Median score at GAS (Q1–Q3)	5 (3–8.8)
Depression (GDS‐SF)	Depressive symptoms	57 (46.7)
	No depressive symptoms	65 (53.3)
	Median score at GDS‐SF (Q1–Q3)	1 (1–2)
Body dissatisfaction (DPG, FRS)	< |2| (No BD)	67 (54.9)
	|2| (Moderate BD)	38 (31.1)
	> |2| (Severe BD)	17 (13.9)
	Median DPG (Q1–Q3)	|1| (|1|–|2|)
Body appreciation (BAS‐2)	Q1	33 (27.0)
	Q2‐3	41 (33.6)
	Q4	48 (39.3)
	Median (Q1–Q3)	38 (31–45)

**Abbreviations**: BA, body appreciation; BAS‐2, body appreciation scale‐2; BD, body dissatisfaction; BMI, body mass index; DPG, desired‐perceived gap; FRS, figure rating scale; GAS‐10, geriatric anxiety scale; GDS‐SF, geriatric depression scale—short form; Q, quartile.

### 3.2 Mental Health Symptoms

The assessment of anxiety symptoms (GAS‐10) revealed that 57% of participants reported no clinically relevant anxiety symptoms, 14% experienced light anxious symptoms, 7% showed moderate anxious symptoms, and 16% presented with severe anxiety symptoms. The median GAS‐10 score was 5, with an interquartile range of 3–8.8. Regarding depression (GDS‐SF), 57% of participants exhibited depressive symptoms, whereas 43% reported no depressive symptoms. The median score for depression was 1, with an interquartile range of 1–2.

### 3.3 Body Perception

BD and BA were assessed using two psychometric tests: FRS and BAS‐2. Analysis of the DPG (derived from the FRS) revealed that 45.1% exhibited BD, with 31.1% showing moderate BD and 13.9% severe BD. 54.9% of participants did not show any BD.

Participants displayed moderate levels of BA, falling between the first and the third quartiles, as indicated by a median BAS‐2 score of 38, with only 39.3% with a BAS‐2 score in the last quartile, indicating high levels of BA.

### 3.4 The Impact of Different BMI Cutoffs on BD and BA

We compared the three different BMI stratifications with BD and BA, as measured by the FRS and BAS‐2 (Table 2). There were significant differences in BD across BMI categories for all three thresholds. The standard BMI stratification showed higher BD levels among subjects with obesity (mean DPG = 2.1) compared to those with body weight within the typical range (median DPG = 1.0). The association persisted also when the 27.27 kg/m^2^ and the 28 kg/m^2^ cutoffs were applied, with a median DPG value of 2 in participants with overweight/obesity compared to participants within the typical range (mean DPG value = 1.2). In contrast, none of the BMI stratifications were significantly associated with BA.

**TABLE 2:** Associations between the three different BMI categorizations and body uneasiness (DPG) / body appreciation (BAS‐2).

A. Associations between BMI (standard groups) and DPG/BAS‐2 (Kruskal‐Wallis test)						
BMI cutoff(kg/m^2^)	Measure	H_(2)_	p	BMI groups	Mean	SD
**Standard**	**DPG**	18.496	< 0.0001	Normal weight	1.023	0.952
				Overweight	1.552	0.940
				Obesity I	2.105	0.994
	**BAS‐2**	2.380	0.304	Normal weight	38.977	8.429
				Overweight	36.052	9.339
				Obesity I	35.684	10.791

B. Associations between BMI (revised cutoffs) and DPG/BAS‐2 (Mann‐Whitney test)						
BMI cutoff (kg/m^2^)	Measure	U	p	BMI groups	Mean	SD
**27.27**	**DPG**	984.000	< 0.0001	Normal weight	1.190	0.935
				Overweight/Obesity	1.930	0.985
	**BAS‐2**	1879.000	0.333	Normal weight	37.861	8.433
				Overweight/obesity	35.767	10.659
**28**	**DPG**	865.500	< 0.0001	Normal weight	1.202	0.967
				Overweight/obesity	2.000	0.900
	**BAS‐2**	1849.000	0.162	Normal weight	38.060	8.447
				Overweight/obesity	35.053	10.758

**Abbreviations**: BAS‐2, body appreciation scale–2; DPG, desired‐perceived gap; Obesity I, obesity first degree; SD, standard deviation; H_(2)_, Kruskal‐Wallis test statistic with 2 degrees of freedom; U, Mann‐Whitney U test statistic; *p*, *p*‐values.

### 3.5 Associations Between BD and BA With Anxious and Depressive Symptoms

A Mann‐Whitney U test was performed to compare symptoms of anxiety and depression in our sample based on BD, with a significant difference between participants with and without BD both for anxiety (mean GAS value of 6.7 vs. 5.1, *p* = 0.044) and depression (mean GDS value of 1.94 vs. 1.49, *p* = 0.026).

For BA, we run a Kruskal‐Wallis test to investigate differences according to the level of body appreciation. No differences were found in depressive symptoms (Kruskal Wallis H (2) = 0.893, *p* = 0.640), whereas different levels of BA showed significantly different degrees of anxious symptoms (Kruskal‐Wallis H (2) = 6.491, *p* = 0.039) with participants in the lowest quartile of BA (median BAS‐2 score = 7.25) showing significantly higher levels of anxiety compared to those in the highest quartile (median BAS‐2 score = 4.06).

## 4 Discussion

This study aimed to explore BD and BA in a community‐based sample of 122 Italian men aged 65 or older. Results showed that 39.3% of participants reported BA, while 45.1% experienced some level of BD and 13.9% reported severe BD. BD was significantly associated with all three BMI thresholds examined (standard, 27.27 kg/m^2^, and 28 kg/m^2^), with participants with BMI in the obesity range reporting higher BD compared to those within the typical weight range. BD was also significantly associated with symptoms of anxiety and depression, with participants reporting BD showing higher levels of both compared to those without BD. In contrast, BMI values were not significantly correlated with BA. Depressive symptoms did not differ significantly across BA levels, while lower BA was associated with higher anxiety levels.

In our study nearly half of the sample reported some degree of BD, and a significant proportion indicated severe dissatisfaction, which is consistent with findings from similar studies on comparable populations (Menezes et al. 2014; Mangweth‐Matzek et al. 2016). Our results demonstrated the considerable impact that BMI thresholds have on BD among older men, revealing that in all three BMI thresholds participants with overweight/obesity reported higher levels of dissatisfaction compared to those in the typical weight category. Parallelly, our results show that individuals classified as normal weight according to the updated BMI thresholds exhibited higher BD compared to those classified as normal weight under the standard BMI criteria. This pattern suggests that internalization of these BMI thresholds may influence how individuals perceive their bodies and self‐worth.

In this context, updating BMI cutoffs for older individuals is primarily a health‐related consideration, given the significant physiological changes that occur with aging (Winter et al. 2014). These changes include alterations in water content, endocrine function, and the distribution of adipose and muscle tissues (Kvamme et al. 2012; Winter et al. 2014), which can influence overall health outcomes such as mortality and general functional status (Bahat et al. 2012; Kvamme et al. 2012; Winter et al. 2014; Sun et al. 2017). Evidence indicates that the optimal BMI range for individuals over 65 may require adjustment to reflect these age‐related physiological shifts, analogous to the tailored BMI thresholds used for children and adolescents (Kıskaç et al. 2022). Beyond health implications, BMI is associated with BD, with higher BMI linked to increased levels of BD even among older men, a group often underrepresented in body image research (Bennett et al. 2020; Steptoe and Frank 2023). Considering the influence of societal standards regarding body image, we hypothesize that updating BMI thresholds could help reduce the dissonance between perceived and ideal body image. This would be particularly relevant for individuals at risk of overweight‐related BD: by aligning BMI classifications with age‐related physiological changes, BMI thresholds could provide a framework that better reflects realistic body compositions for this population.

Our results regarding BA indicate that this factor appears to be disconnected from BMI. Our findings align with recent research by Zhang (2022), which did not find significant associations between BMI and BA in a large sample of older Chinese men (Zhang et al. 2022). Conversely, a recent large meta‐analysis found that the association between BA and BMI was negative and small‐moderate for females and negative and small for males (He et al. 2020). Authors suggested that culture and ethnicity could potentially moderate the association for both genders (He et al. 2020). To consider other factors that may influence BA, such as cultural background, Hanson (2024) found that BA remained stable across age groups in women from White Western, Black Nigerian, and Chinese cultures (Hanson et al. 2024). Nigerian women reported the highest BA and Western women had the lowest. The study also highlighted that higher internalization of thinness or athletic ideals and greater perceived sociocultural pressure were associated with lower BA (Hanson et al. 2024). This regarded all cultures and age groups (Hanson et al. 2024). Such evidence suggests that concerns about body perception are closely tied to societal standards besides actual body shape and size (Cohen et al. 2019).

Concerning mental health symptoms, participants with BD exhibited significantly higher levels of anxiety and depression compared to those without BD. These findings are consistent with previous research reporting the negative psychological consequences of BD: different studies have shown that individuals with higher levels of BD experience increased mental distress (Cash 2004; Rakhkovskaya and Holland 2017; Moradi et al. 2022). Across different levels of BA, depressive symptoms did not vary significantly, but we found a notable inverse association between BA and anxiety levels. Also, this aspect aligns with existing literature suggesting that body image issues are more strongly correlated with anxiety than with depression, possibly due to the constant worry and distress associated with appearance concerns (Kim and Kang 2015; Ramseyer Winter et al. 2019; Ramseyer Winter et al. 2020). In particular, individuals with lower BA may be more vulnerable to experiencing higher anxiety as they struggle to meet societal standards of appearance, which can exacerbate feelings of insecurity and self‐criticism (Gendron and Lydecker 2016).

Moreover, elevated levels of BD alongside lower BA are associated with a higher likelihood of developing EDs (Tylka 2004; Lantz et al. 2018; Dal Brun et al. 2024). Despite growing attention in recent years (Rohde et al. 2015; Kostecka et al. 2019; Manzato et al. 2022), ED's prevalence remains often underdiagnosed in men (Hudson et al. 2007; Smink et al. 2012; Raevuori et al. 2014). Most studies examining BD as a risk factor for EDs have focused primarily on younger males, with limited attention given to older males (Speciani et al. 2021). This underrepresentation contributes to the underestimation of EDs in adulthood and older age (Podfigurna‐Stopa et al. 2015; Conceição et al. 2017). This lack of attention can be attributed to the media's stereotypical portrayal of EDs as disorders that primarily affect adolescent women, in addition to the lower visibility of older men and the prevalence of ageism, which perpetuates age‐discriminatory attitudes and practices (Butler 1969; Ribeiro et al. 2018; Lyons et al. 2018). Recent epidemiological data highlight that men of all ages are vulnerable to body image concerns, making them susceptible to developing EDs even later in life (Manzato et al. 2022). This is supported by few existing studies reporting notable prevalence rates of ED symptoms and EDs in middle‐aged and older men (McManus et al. 2009; Mangweth‐Matzek et al. 2016). Together, these observations point to the importance of examining BD and BA in older men, and how these relate to BMI and psychological symptoms in order to better understand factors contributing to EDs in this population.

This study comes with some strengths and limitations. Among its strengths, it investigated BD and BA in a specific and under‐researched demographic, and included a diverse age range within the elderly population, incorporating very elderly individuals, a group that is particularly challenging to recruit in research studies. Nonetheless, several limitations warrant consideration. First, the small, racially homogeneous sample of older Caucasian men may limit the generalizability of our findings. Second, the high educational attainment of our participants might have introduced a bias, potentially affecting the representation of individuals with different educational backgrounds. Further studies are needed with a larger and more diverse sample to enable examination of potential confounding factors, including age, educational level, and physical activity. A

Further limitation is that the study relied on self‐administered questionnaires and self‐reported measures of height and weight. Such self‐reported data can be prone to measurement error and social desirability bias, particularly in older adults, which may affect the accuracy of BMI calculations and should be taken into account when interpreting the results. Moreover, FRS and BAS‐2 are well‐known instruments to evaluate body experience but the use of scales specifically tailored for assessing body uneasiness in the elderly would have ensured more consistency and comparability. Finally, our study did not include direct measures of body composition, such as bioimpedance analysis or dual‐energy X‐ray absorptiometry (DXA) scans, which limits the ability to precisely assess fat distribution, lean mass, and age‐related changes. Without these measures, conclusions regarding the appropriateness of BMI thresholds for older adults may be considered preliminary.

In conclusion, this study reports a significant prevalence of BD in a sample of Italian men over 65, with nearly half of the participants reporting some level of dissatisfaction strongly associated with BMI thresholds, particularly in individuals with overweight/obesity. Less than half of the sample reported BA, and it did not appear to be significantly influenced by BMI, consistent with previous literature suggesting that factors such as cultural and social body ideals play a more significant role in structuring self‐appreciation. Our results highlight the psychological implications of BD, showing a connection with higher levels of anxiety and depression, and BA, which is associated only with anxiety. These findings points to the importance of evaluating BD and BA in older men and examining related mental health outcomes. While these data are preliminary, they suggest that promoting age‐specific BMI thresholds could help reduce the internalized criticism associated with body image problems. Indeed, by aligning BMI thresholds with the physiological changes that occur with aging, we could mitigate the disconnect between perceived and ideal body image. This change could lead to a more positive self‐image and decrease the psychological distress often linked to societal body ideals, potentially contributing to improved self‐perception and mental health in this population.

## Author Contributions

**Alfonso Martone**: conceptualization, methodology, formal analysis, investigation, data curation, writing–original draft preparation. **Silvia Tempia Valenta**: methodology, investigation, data curation, writing–original draft preparation. **Federica Marcolini**: investigation. **Diana De Ronchi**: supervision. **Emilia Manzato**: conceptualization, writing–review and editing. **Anna Rita Atti**: conceptualization, writing–review and editing. All authors have read and approved the final manuscript.

## Funding

The authors have nothing to report.

## Conflicts of Interest

The authors have no relevant financial or non‐financial interests to disclose.

## Ethics Statement

This study was conducted in accordance with Italian privacy law, specifically the Code on the Protection of Personal Data (Legislative Decree No. 196/2003), as amended by Legislative Decree No. 101/2018, and complied with the ethical principles of the Declaration of Helsinki. The statistical evaluation of collected data was carried out after complete anonymization.

## Data Availability

The dataset analyzed during the current study is available from the corresponding author upon reasonable request.
